# How to Approach Elevated NT-pro BNP Level on Admission to Prevent Left Ventricular Aneurysm Following Acute ST-Segment Elevation Myocardial Infarction

**DOI:** 10.36660/abc.20190773

**Published:** 2020-02

**Authors:** Teruhiko Imamura

**Affiliations:** University of Toyama, Toyoma - Japan

**Keywords:** Heart Failure/physiopathology, Natriuretic Peptide, B-Type, ST Elevation Myocardial Infarction, Coronary Aneurysm/complications, Stroke Volume, Indicators of Morbidity and Mortality

I have read with great interest the article written by Celebi and colleagues,^1^ demonstrating that elevated plasma N-terminal pro-B-type natriuretic peptide (NT-pro BNP) level on admission is a significant predictive biomarker of development of left ventricular aneurysm following acute ST-segment elevation myocardial infarction (STEMI) in the current era.

Their findings are straightforward and would lead us the next concerns. The first concern is reversibility of NT-pro BNP and its impact on post-STEMI outcomes. Is the reduction of NT-pro BNP following STEMI associated with a lower rate of left ventricular aneurysm formation?

Another concern is the methodology to improve NT-pro BNP. The uses of several medications including P2Y12 inhibitors were associated with the avoidance of left ventricular aneurysm. However, given the retrospective nature of their study, they cannot exclude the confusion between the use of medications and the severity of STEMI. Prospective studies are warranted to investigate the implication of aggressive interventions using mechanical cardiac unloading (for example, Impella) or medical cardiac unloading (for example, aggressive dose-titration of beta-blocker) on the prevention of left aneurysm formation post-STEMI.
