# Supplementary material for: How to Approach Elevated NT-pro BNP Level on Admission to Prevent Left Ventricular Aneurysm Following Acute ST-Segment Elevation Myocardial Infarction
Source: Arq Bras Cardiol. 2020 Feb;114(2):323–4. doi: 10.36660/abc.20190773 (PMC7077570; doi:10.36660/abc.20190773)
Supplement: Related texts

# Reply

**Authors:** Savas Celebi, Ozlem Ozcan Celebi, Berkten Berkalp

**Dear Editor,**

We would like to thank the authors for their interest in our study.

The authors raised important future aspects about the findings of our study.^1^ Regarding our study results, there is a relationship between N-terminal pro-B-type natriuretic peptide (NT-pro BNP) level and LV (left ventricular) aneurysm formation. However, further studies are needed to address the details of this relationship.

Our study did not include serial measurement of NT-pro BNP.^1^ Therefore, we do not have any relevant answer for the reversibility of NT-pro BNP. However, this may be an interesting approach during myocardial infarction regarding LV aneurysm formation.

Medical or mechanical interventions on the prevention of LV aneurysm are another debate. In the IABP Shock trial, the authors did not determine a significant difference between the groups treated with IABP and without IABP concerning NT-pro BNP levels.^2^ In contrast, they determined a significant improvement of B-type natriuretic peptide (BNP) levels using IABP. We agree that further prospective trials are needed to determine the association between the methods of reducing NT-pro BNP or BNP and LV aneurysm formation.

## References

[1] Celebi S, Celebi OO, Cetin S, Cetin HO, Tek M, Gokaslan S (2019). Usefulness of admission plasma NT –pro-BNP Level to Predict Left Ventricular Aneurysm Formation after Acute ST-Segment Elevation Myocardial Infarction. Arq Bras Cardiol.

[2] Lemm H, Prondzinsky R, Geppert A, Russ M, Huber K, Werdan K (2010). BNP and NT-proBNP in patients with acute myocardial infarction complicated by cardiogenic shock: results from the IABP Shock trial. Crit Care.
